# HIV-1 Tat Activates Neuronal Ryanodine Receptors with Rapid Induction of the Unfolded Protein Response and Mitochondrial Hyperpolarization

**DOI:** 10.1371/journal.pone.0003731

**Published:** 2008-11-14

**Authors:** John P. Norman, Seth W. Perry, Holly M. Reynolds, Michelle Kiebala, Karen L. De Mesy Bentley, Margarita Trejo, David J. Volsky, Sanjay B. Maggirwar, Stephen Dewhurst, Eliezer Masliah, Harris A. Gelbard

**Affiliations:** 1 Center for Neural Development and Disease, the University of Rochester School of Medicine and Dentistry, Rochester, New York, United States of America; 2 Department of Neurology (Child Neurology Division), the University of Rochester School of Medicine and Dentistry, Rochester, New York, United States of America; 3 Department of Environmental and Health Sciences, the University of Rochester School of Medicine and Dentistry, Rochester, New York, United States of America; 4 Department of Laboratory Medicine and Pathology, the University of Rochester School of Medicine and Dentistry, Rochester, New York, United States of America; 5 Department of Microbiology and Immunology, the University of Rochester School of Medicine and Dentistry, Rochester, New York, United States of America; 6 Molecular Virology Division, St. Lukes-Roosevelt Hospital Center, Columbia University, New York, New York, United States of America; 7 Department of Neurosciences and Department of Pathology, School of Medicine, University of California San Diego, La Jolla, California, United States of America; Federal University of Sao Paulo, Brazil

## Abstract

Neurologic disease caused by human immunodeficiency virus type 1 (HIV-1) is ultimately refractory to highly active antiretroviral therapy (HAART) because of failure of complete virus eradication in the central nervous system (CNS), and disruption of normal neural signaling events by virally induced chronic neuroinflammation. We have previously reported that HIV-1 Tat can induce mitochondrial hyperpolarization in cortical neurons, thus compromising the ability of the neuron to buffer calcium and sustain energy production for normal synaptic communication. In this report, we demonstrate that Tat induces rapid loss of ER calcium mediated by the ryanodine receptor (RyR), followed by the unfolded protein response (UPR) and pathologic dilatation of the ER in cortical neurons *in vitro*. RyR antagonism attenuated both Tat-mediated mitochondrial hyperpolarization and UPR induction. Delivery of Tat to murine CNS *in vivo* also leads to long-lasting pathologic ER dilatation and mitochondrial morphologic abnormalities. Finally, we performed ultrastructural studies that demonstrated mitochondria with abnormal morphology and dilated endoplasmic reticulum (ER) in brain tissue of patients with HIV-1 inflammation and neurodegeneration. Collectively, these data suggest that abnormal RyR signaling mediates the neuronal UPR with failure of mitochondrial energy metabolism, and is a critical locus for the neuropathogenesis of HIV-1 in the CNS.

## Introduction

Infection of the central nervous system (CNS) with the human immunodeficiency virus type 1 (HIV) occurs rapidly after primary infection [1]. The phenotype of HIV associated dementia (HAD) after the introduction of highly active antiretroviral therapy (HAART) has changed considerably with a more indolent time course, frequently characterized by waxing and waning neurologic deficits, suggesting a change in nomenclature to HIV-1 associated neurologic deficits (HAND) [2]. Alarmingly, more recent studies of incidence and prevalence of the neurologic component of HIV-1 infection demonstrate that neural injury continues in some patients regardless of the ability of HAART to achieve virologic suppression and normalization of immunologic parameters [3]. The CNS can act as a reservoir for HIV as agents that comprise HAART do not achieve a level of CNS penetration that can fully eradicate the virus [2], [4]. In patients with improved systemic health from HAART, it is HAART's failure to control HIV-1's effects on the signaling pathways that mediate normal communication between immune effecting glias and vulnerable neurons, that has substantially contributed to the rise in HAND prevalence since 2000 [5]. Thus HAND continues to be a problem of pandemic proportions.

Since HIV-1 only infects CNS cell types that express the chemokine receptors CD3, CCR5 and/or CXCR4 (i.e. microglia, perivascular macrophages, and a restricted population of astrocytes) [6], structural damage with accompanying neurologic disease [7] occurs because of pathway activation that leads to release of inflammatory molecules such as nitric oxide (NO), tumor necrosis factor alpha (TNF-α), and platelet activating factor (PAF); changes in ambient recycling of glutamate by astrocytes; and the release of viral regulatory proteins, such as the trans activator of transcription protein (Tat) [8]–[11] and the envelope protein gp120 [12]. At the light microscopic level, the neuropathology of HIV-1 infection is notable for changes in the dendritic arbor with varicosities (“beading”–13); accumulation of beta amyloid precursor protein (β-APP) in axons [14]; neuronal apoptosis [15], [16]; and reactive astrocytosis, microgliosis, and multinucleated giant cells [17]. Surprisingly, ultrastructural analyses of brain tissue from patients with HIV-1 infection have focused on changes in endothelial architecture, including thinning and vacuolization of the basal lamina [18], but no study has focused on changes in intracellular organelles or synaptic architecture of neurons.

Of the many HIV-induced neurotoxins, Tat is remarkable because it is actively released into the extracellular space by infected microglia, macrophages and astrocytes [10; 19–21]. Unbound Tat has been detected in the sera of HIV^+^ patients, reaching concentrations as high as 40 ng/mL [22]. It should be noted that this measurement is probably a gross underestimate of Tat's local concentration; Tat *in vivo* can be sequestered by endogenous glycosaminoglycans and heparin sulfates effectively lowering the detectable amounts of Tat circulating unbound. This observation lends credence to the notion that infiltrating microglia/macrophage adjacent to a synapse would have greatly increased local concentrations of Tat. However, once released from a cell Tat can enter virtually all neural cell types via its arginine-rich basic domain, termed the protein transduction domain (PTD) [23]–[26].

Tat can modulate intracellular calcium concentrations through activation of endoplasmic reticulum (ER) pathways in vulnerable neurons [26]–[28]. Protein folding in the ER relies on foldases, chaperones, and lectins that require high concentrations of calcium and an oxidized environment in order to perform properly (Schroder 2005, Wetmore 1996). This in turn raises the question of whether Tat can overwhelm the protein folding capacity of the ER and induce the unfolded protein response (UPR) pathway [29].

Induction of the UPR pathway is designed to reduce net protein translation and results in the up-regulation of a specific set of genes that function to relieve this stress. Phosphorylation of the transmembrane protein kinase–like endoplasmic reticulum kinase (PERK) is one of the initial events in the UPR pathway and is responsible for the downstream phosphorylation of eukaryotic initiation factor 2α (eIF2α) that prevents 80S ribosome assembly, inhibiting protein translation [30]. Inositol requiring kinase 1 (IRE1) can dimerize in conjunction with PERK phosphorylation and cleave the mRNA of X-box binding protein 1 (XBP1) to produce an active 54-kDa transcription factor that is responsible for maintaining the UPR pathway [31]–[33]. If the offending ER toxicant is eliminated, the UPR pathway shuts down and normal protein translation and folding resumes. Conversely, if the UPR pathway remains functionally active, the pro-apoptotic protein CHOP (CCAAT/enhance binding protein (C/EBP) homologous protein) is up-regulated and the cell undergoes apoptosis [34].

Our laboratory and others have previously described the phenomenon of mitochondrial hyperpolarization in cortical neurons after exposure to Tat [35] and other stressors [36]–[38]. Previously, we demonstrated that application of Tat to cortical neurons induced a rapid decrease of mitochondrial calcium leading us to speculate that it was loss of the free calcium cation from mitochondria that resulted in hyperpolarization of mitochondrial membrane potential (ΔΨ_m_) [39]. Due to the importance of the ER in both calcium signaling and mitochondrial function, we investigated the effect of HIV-1 Tat on sequestration of calcium in the ER and demonstrate that HIV-1 Tat induces the rapid loss in ER calcium through the activation of the ryanodine receptor (RyR) with initiation of the UPR. We further show that antagonism of the RyR reversed Tat-induced hyperpolarization of ΔΨ_m_. *In vivo* delivery of HIV-1 Tat to murine CNS also results in pathologic dilation of ER and changes in mitochondrial morphology. Furthermore, these ultrastructural changes also occur in neurons of the frontal cortex from patients with HIV-1 encephalitis and dementia. These results suggest a common mechanism via RyR signaling that rapidly initiates endoplasmic reticulum and mitochondrial calcium release as part of a generalized neuronal stress response that appears to have enduring consequences for the neuropathogenesis of HIV-1.

## Results

### Acute exposure to Tat induces calcium loss from the ER via the ryanodine receptor

In our previous studies of cortical neurons exposed to HIV-1 Tat, we concluded that neuronal mitochondria suffered a loss of energy metabolism reflected by decreased NAD(P)H, as well as [Ca^+2^][39]. Because of the changes in mitochondrial [Ca^+2^], we investigated the ER as a potential locus for these effects. The ER is responsible for the storage of Ca^2+^ and has the ability to induce rapid efflux of Ca^2+^ in response to a variety of cellular signals, including inositol 1,4,5-triphosphate (IP_3_) receptors and ryanodine receptors (RyR) [40]. There are several dyes used to measure intracellular Ca^2+^ concentration; however none are specific to the ER [41]. Using a ratiometric, ER-targeted calmodulin CFP:EYFP (cyan fluorescing protein to enhanced yellow fluorescing protein) construct, we were able to measure ER calcium concentrations in real time after application of HIV-1 Tat to cortical neurons [42]. Since Tat is known to induce apoptosis in neurons in a dose-dependent fashion, for all subsequent experiments we used the lowest sub-lethal concentration of Tat (100 ng/ml = [∼8 nM]) that would allow us to reproducibly model neuronal dysfunction, but not apoptosis [28], [35].

**Figure 1:**
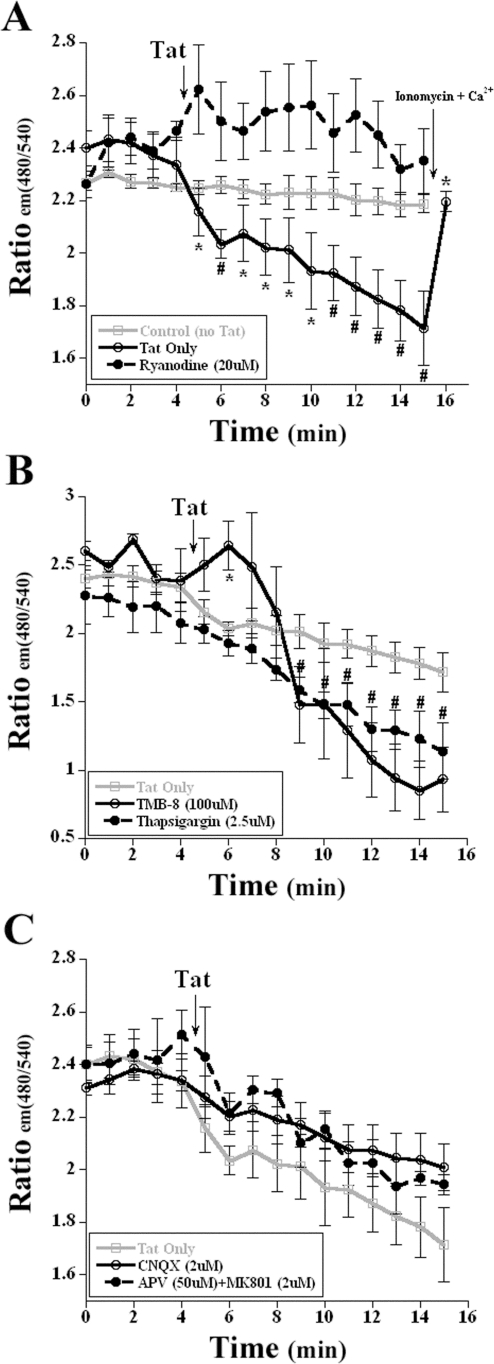
Endoplasmic reticulum calcium decrease via RyR in response to HIV-1 Tat. A, Application of 8 nM Tat to transfected neurons induced the loss of the CFP:EYFP fluorescent signal, indicating a loss in ER calcium (labeled ‘Tat Only’). Pretreatment with ryanodine for 30 min prior to exposure inhibited this loss in ER calcium. To ensure that the loss in fluorescence was not due to overexposure, a photo-bleaching control, Control (no Tat), was performed to demonstrate the stability of the fluorescent signal. Application of the positive controls, ionomycin (2 µM) or calcium (10 mM) increased the ER CFP:EYFP fluorescence (n = 5; *, p<0.05; #, p<0.01). B, Pretreatment with the IP_3_ inhibitor TMB-8 and the sarco-endoplasmic reticulum Ca^2+^-ATPase inhibitor thapsigargin failed to attenuate the loss of ER calcium when the transfected cortical neurons were exposed to 8 nM Tat. (n = 4; *, p<0.05 for TMB-8; #, p<0.05 for thapsigargin). C, Transfected neurons that were pretreated with either APV/MK-801 or CNQX exhibit the same relative loss of the ER CFP:EYFP fluorescence, demonstrating the loss of calcium from the ER and not influx from activation of ionotropic glutamate receptors (n = 4). In Figure 1A, the mean values of CFP:EYFP fluorescence for ‘Tat Only’ treatment group were plotted, and treated time points were compared with control time points (before addition of Tat) for statistical significance. For all other groups (Figure 1A–C), the values of CFP:EYFP fluorescence were plotted and all time points were compared with the treatment group ‘Tat Only’ time points for statistical significance.

Cortical neurons exposed to Tat exhibited a rapid loss of ER Ca^2+^stores as indicated by the loss in CFP:EYFP fluorescence (Figure 1). FRET imaging has an advantage over traditional calcium sensitive dyes in that the ratio between the two fluorophores acts as an internal control and is less susceptible to fluctuations and photo-bleaching that commonly plague single wavelength dyes. Exposure to 8 nM Tat elicited an initial loss of ∼6% of the fluorescent signal with a continued decrement to ∼80% of control fluorescence over a 10 minute period (Figure 1A). To investigate the mechanism responsible for the release of ER Ca^2+^, we pre-treated cortical neurons for 30 min with 20 µM ryanodine (Ry), a concentration which inhibits RyR channel opening (Bardo 2006). Tat-challenged cells showed no loss of ER CFP:EYFP fluorescence when pre-treated with ryanodine, indicating that ryanodine blocks Tat-induced destabilization of the ER Ca^2+^ pool (Figure 1A).

There are several other mechanisms that can mobilize ER Ca^2+^ pools, thus altering [Ca^2+^] in this organelle, including IP_3_-sensitive stores and the sarco-/endoplasmic reticulum Ca^2+^–ATPase (SERCA) pump [40]. To determine whether either of these mechanisms was responsible for the observed changes in ER [Ca^2+^], we pretreated cortical neurons for 30 minutes with either 100 nM of the IP_3_ inhibitor TMB-8 or with 2.5 µM of the SERCA pump inhibitor thapsigargin (Figure 1B). When compared to neurons exposed to only Tat, TMB-8 and thapsigargin failed to attenuate the loss in ER [Ca^2+^] (Figure 1B). In fact, inhibition of the SERCA pump accentuated the Ca^2+^ loss, most likely by rendering the SERCA pump unable to sequester Ca^2+^ back into the ER (Figure 1B, Ref. 40).

Tat can also activate N-methyl-D-aspartic acid receptor (NMDA-R) and non-NMDA glutamate receptors (GluR) [43], [44]. To rule out the possibility of these interactions, pharmacological antagonists of the NMDA receptor recognition site and ion channel were utilized in combination (Figure 1C). Pretreatment (30 min) with 50 µM of APV and 2 µM of MK-801 failed to attenuate the loss of ER Ca^2+^ (Figure 1C). Likewise, to eliminate the possibility of AMPA-R activation that might contribute to excess excitatory neurotransmission, neurons were pretreated with 2 µM of the AMPA and kainate receptor antagonist CNQX, which also failed to inhibit the loss of ER Ca^2+^ (Figure 1C). The addition of the positive control ionomycin and 10 mM of Ca^2+^, increased the absolute magnitude of CFP:EYFP fluorescence, indicating an increase in ER [Ca^2+^] as expected (Figure 1A).

### Unfolded Protein Response pathway proteins are induced by HIV-1 Tat

A consequence of rapid calcium loss from the ER is the induction of the UPR, a signaling pathway that can regulate the volume of the ER to accommodate an increase in unfolded proteins [45]. Because of the results depicted in Figure 1, we investigated whether Tat could induce changes in protein species involved in this response.

**Figure 2:**
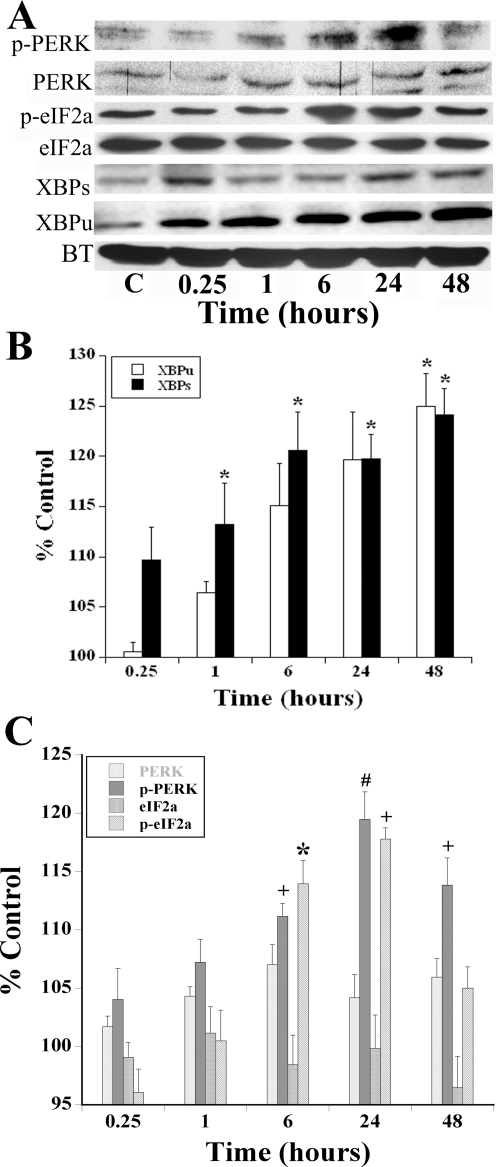
Endoplasmic Reticulum UPR pathway proteins are up-regulated by HIV-1 Tat. A, The treatment of cortical neurons with Tat (8 nM) resulted in the increase in activation of several UPR proteins, including either phosphorylated species or an increase in the active protein. There was an increase in p-PERK, eIF2α, XBP_u_ and XBP_s_ levels of protein in response to Tat exposure. The panels depicting p-PERK, PERK, p-eIF2a, eIF2a and α-tubulin (AT) are from one experimental replicate while the panels depicting XBPs, XBPu are from another experiment. Expression of AT remained invariant after exposure to Tat for all experimental replicates. B, Using densitometry, the relative expression of each of the UPR proteins were examined with all bands normalized to ΑΤ to demonstrate that equal amounts of protein were loaded into each lane. The relative abundance of XBP species are shown in Panel B and PERK and eIF2α species are shown in Panel C. The phosphorylated PERK and eIF2α proteins increased to 119% and 117% control respectively when exposed to Tat for 24 hours (N = 7, #, p<0.01; +, p<0.005). Both XBP_u_ and XBP_s_ levels demonstrated a similar increase in concentrations during the described time course (n = 6; *, p<0.05).

In response to 8 nM Tat treatment, a dose that is sub-lethal, there was a qualitative increase in p-PERK and p-eIF2a between 6–24 hours that persisted for 48 hours as detected by immunoblotting (Figure 2A) [27], [28], [35], [43], [44], and could be blocked by co-incubation with antagonist doses of ryanodine (Supplemental Figure S1). Densitometric analyses confirmed these changes as statistically significant (Figure 2C), even though the absolute magnitude of changes was relatively modest when averaged across 6 experimental replicates. Additionally, there was both a total increase in XBP1 (XBP_u_+XBP_s_) protein expression as well as a 25% increase in the active XBP_s_ isoform (Figure 2A, B). The positive control for induction of the UPR, tunicamycin (1 µg/ml for 6 h), increased the relative abundance of phosphorylated species of PERK and eIF2α, as well as XBP1 expression in a manner similar to that of Tat (data not shown). The data taken together indicate that exposure to a sub-lethal dose of Tat up-regulates the UPR at the protein level within 15 minutes and persists for at least 48 hours.

### HIV-1 Tat induces ER morphological pathology in cortical neurons

**Figure 3:**
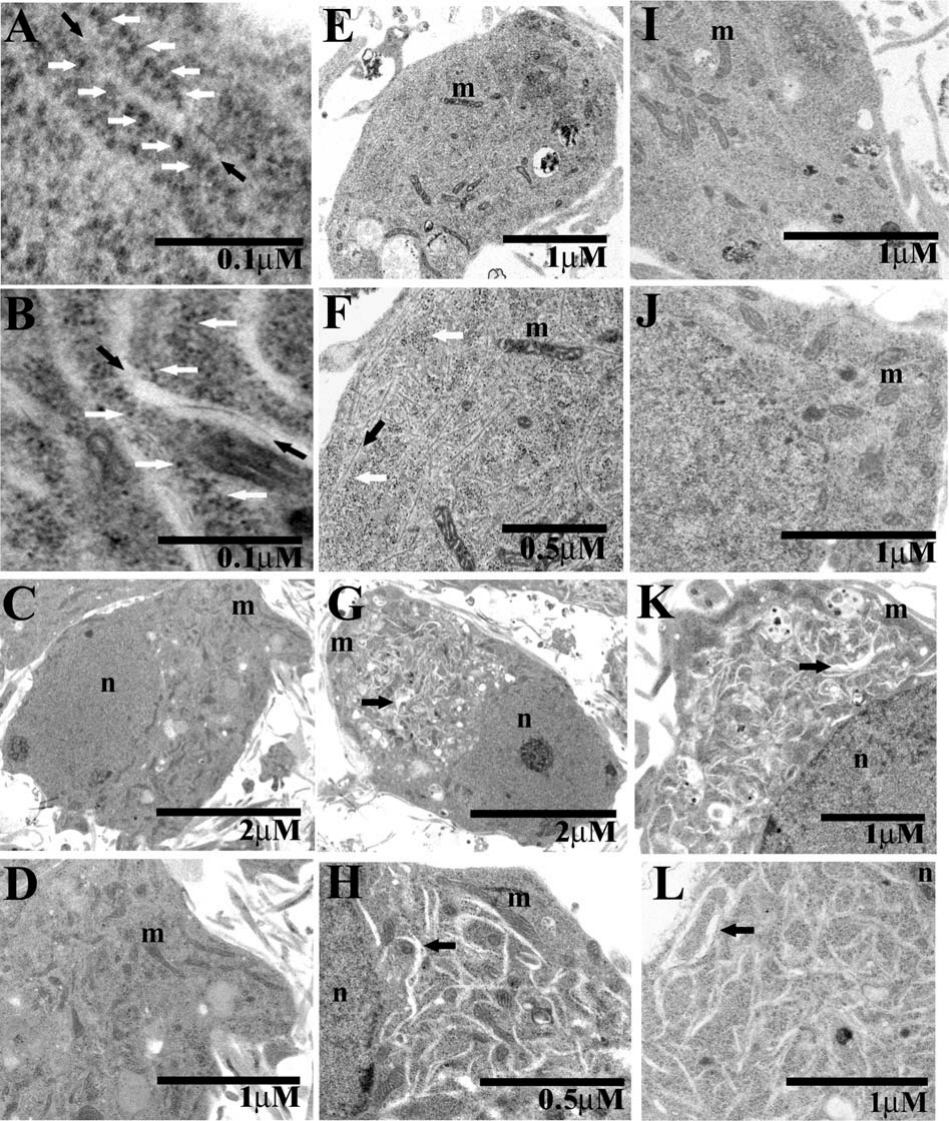
HIV-1 Tat induces endoplasmic reticulum neuropathologic changes in cortical neurons. A–L, The following symbols are used throughout the montage: m, mitochondria; n, nucleus; black arrows, ER; white arrows, ribosomes. A, Control neurons displayed classic rough ER morphology with ribosomes contiguous with the rough ER membrane. B, Cortical neurons treated with 8 nM Tat demonstrated a dilation of ER and a dissociation of ribosomes. C, An untreated cortical neuron displayed normal morphology. D, A magnified image of the untreated cortical neuron in C. E, Cortical neuron treated with 8 nM Tat for 15 minutes. F, A magnified image of the treated [8 nM Tat] cortical neuron. G, Cortical neuron treated with [8 nM] Tat for 15 minutes displayed dilated ER. H, Magnified image of treated neuron in G. The dilated ER at this level of magnification was highly prominent. I, Cortical neurons pretreated with an antagonist concentration [20 µM] of ryanodine for 30 min before [8 nM] of Tat treatment displayed none of the pathology demonstrated in previous images. J, Cortical neurons pretreated with the RyR antagonist [25 µM] dantrolene for 30 min before [8 nM] of Tat treatment had similar morphologies to those that received [20 µM] ryanodine pretreatment. K, Cortical neurons pretreated with the IP_3_ inhibitor [100 µM] TMB-8 for 1 hour before Tat [8 nM] treatment failed to attenuate the morphological pathology induced by Tat. L, Cortical neurons pretreated for 30 minutes with 2 µM MK-801 also failed to attenuate the pathology elicited by treatment of Tat [8 nM].

**Figure 4:**
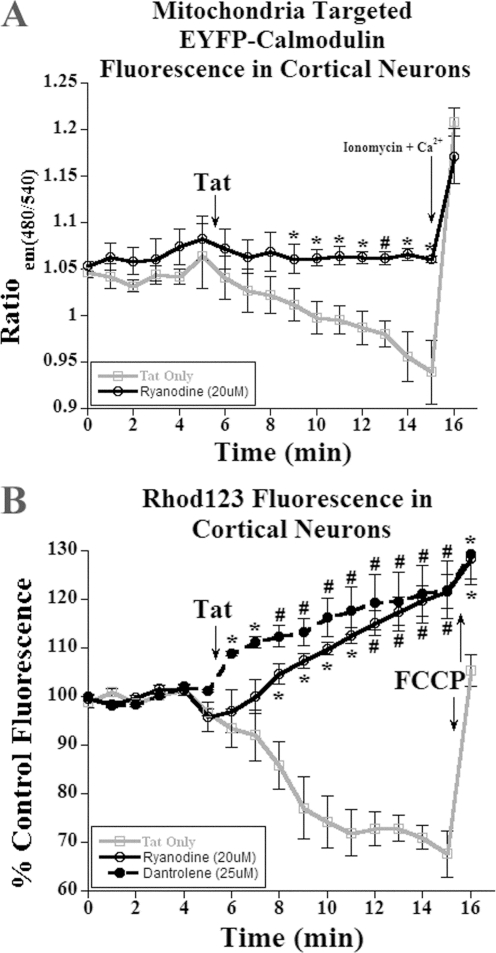
HIV-1 Tat mediated mitochondrial calcium loss and hyperpolarization is mediated through the ryanodine receptor. A, Treatment with 8 nM Tat of cortical neuron in only media resulted in a 10% loss in CFP:EYFP calmodulin fluorescence. The pretreatment with ryanodine for 30 minutes prior to exposure to 8 nM Tat inhibited the loss of fluorescent signal (n = 4; *, p<0.05; #, p<0.01). B, Treatment with 8 nM Tat of cortical neuron resulted in the rapid loss in rhod123 fluorescence, indicating mitochondrial hyperpolarization. Incubation with either an inhibitory concentration of ryanodine or the RyR inhibitor dantrolene for 30 minutes before Tat treatment [8 nM] resulted in the rapid loss in rhod123 signal, indicating mitochondrial depolarization (n = 5; *, p<0.05; #, p<0.001). Addition of the mitochondria specific protonophore, carbonyl cyanide-4(trifluoromethoxy)phenylhydrazone (FCCP; [10 µM]), initiated a rapid depolarization of the mitochondria and served as a positive control. The values of CFP:EYFP and rhod123 fluorescence were plotted and all time points were compared with the treatment group ‘Tat Only’ time points for statistical significance.

We next investigated whether Tat could also induce morphologic features associated with ER stress at an ultrastructural level in our *in vitro* model of cortical neurons. Normal subcellular morphological features of organelles can be seen in cortical neurons treated with control vehicle (Figure 3C, D), including the nucleus, mitochondria and the ER (Figure 3A, C, D). When cortical neurons were treated with 8 nM Tat for 10 minutes, several striking morphological changes occurred (Figure 3E, F). The ER began to increase in abundance and dilate, tubules became clearly visible, and some of the ribosomes were no longer apposed to the ER membrane (Figure 3E, F). After 15 minutes of Tat exposure (Figure 3G, H), the ER underwent labyrinthine dilatation throughout the cytoplasm of affected cortical neurons (Figure 3H). Ribosomes in untreated cells were typically contiguous with the ER membrane (Figure 3A), however after 15 minutes of treatment, Tat also induced ribosomal dissociation, a classic morphologic feature associated with the UPR (Figure 3B; Refs. 48,49). These morphologic changes were disproportionately amplified, but occurred contemporaneously with increases in protein species associated with the UPR (Figure 2) for as yet unclear reasons. Ribosomal dissociation occurs in order to stop new protein synthesis and is another cellular defense mechanism to relieve stress on the ER [50]. Interestingly, mitochondrial morphology remained normal during Tat exposure in our *in vitro* model (Figure 3C, E, G). We next utilized several pharmacologic strategies to attenuate the pathologic appearance of the ER after Tat treatment. Antagonism of the RyR, either with 20 µM Ry or 25 µM dantrolene (Figure 3I and J respectively), completely abrogated the dilation of the ER and ribosomal dissociation. In contrast, the IP_3_ inhibitor TMB-8 and the NMDA-R antagonist MK-801 failed to attenuate the dilation of the ER, further confirming our hypothesis that the RyR is the pathologic locus for Tat-mediated activation of the UPR (Figure 3K and 4L respectively). This finding was buttressed by the demonstration of RyR localization to the cisternae of RER using immunogold labeling (data not shown).

### Mitochondrial ΔΨ_m_ and Ca^2+^ modulated by RyR activation by Tat

We have previously demonstrated that the mitochondrial membrane potential [ΔΨ_m_] and [Ca^2+^] are directly modulated by exposure of cortical neurons to Tat [35], [39]. In our previous study, we speculated that loss in mitochondrial Ca^2+^ results in the observed ΔΨ_m_ hyperpolarization as measured by rhod123 fluorescence [39]. Several laboratories have demonstrated that mitochondria express RyR in addition to ER, but the biologic effects of RyR signaling in mitochondria remain unclear. Because we had observed similar kinetics of Tat-mediated agonism of RyR in ER with Ca^2+^ loss as well as a decrease in mitochondrial [Ca^2+^], we investigated whether Tat-mediated agonism of mitochondrial RyR was responsible for hyperpolarization of ΔΨ_m_ [51], [52].

To examine this question, we transfected cortical neurons with a CFP:EYFP calmodulin construct with a mitochondrial localization sequence to allow us to visualize mitochondrial calcium ([Ca^2+^]_mito_) [39], [53]. After treatment of cortical neurons with 8 nM Tat, we observed a loss in CFP:EYFP fluorescence, confirming a decrease in [Ca^2+^]_mito_ (Figure 4A). When we pre-treated the neurons with an antagonist concentration [20 µM] of ryanodine for 30 min, the loss of [Ca^2+^]_mito_ was significantly attenuated (Figure 4A). Unfortunately, imaging of dantrolene-treated cultures in this paradigm was unsuccessful because of its autofluorescent properties at both the CFP and EYFP wavelengths, which precluded our ability to corroborate whether it also antagonized RyR in this paradigm. The addition of either a positive control, ionomycin or calcium [10 mM] increased the absolute magnitude of CFP:EYPF fluorescence, demonstrating the specificity of mitochondrial RyR effects on [Ca^2+^]_mito_ stores (Figure 4A).

To correlate the loss of [Ca^2+^]_mito_ with mitochondrial hyperpolarization, we used the lipophilic dye rhod123 in this experimental paradigm. Because rhod123 is cationic, it selectively accumulates in the mitochondria matrix proportional to the electronegativity of the mitochondrial membrane potential and thus provides a quantitative measure of ΔΨ_m_. Since rhod123 exhibits auto-quenching properties at high concentrations, a decrease in the fluorescence indicates a more hyperpolarized ΔΨ_m_. Addition of 8 nM Tat to cortical neurons resulted in a loss of rhod123 fluorescent signal, indicating mitochondrial hyperpolarization (Figure 4B). When Tat-challenged neurons were pre-treated with 20 µM Ry for 30 min in order to block the observed [Ca^2+^]_mito_ loss, we observed a significant rise in the rhod123 fluorescence, indicating mitochondrial depolarization. Pretreatment (30 min) with the RyR antagonist, dantrolene, was also tested to further confirm specificity of the RyR for mediating these pathologic effects. A rise in rhod123 signal was also observed with co-incubation of Tat with dantrolene, indicating mitochondrial depolarization (Figure 4B). In aggregate, these data suggest that blocking the RyR inhibits [Ca^2+^]_mito_ loss, and that it is this loss in [Ca^2+^]_mito_ that is responsible for the observed mitochondrial hyperpolarization when neurons are exposed to Tat.

### Localization of RyR on inner mitochondrial membrane

**Figure 5:**
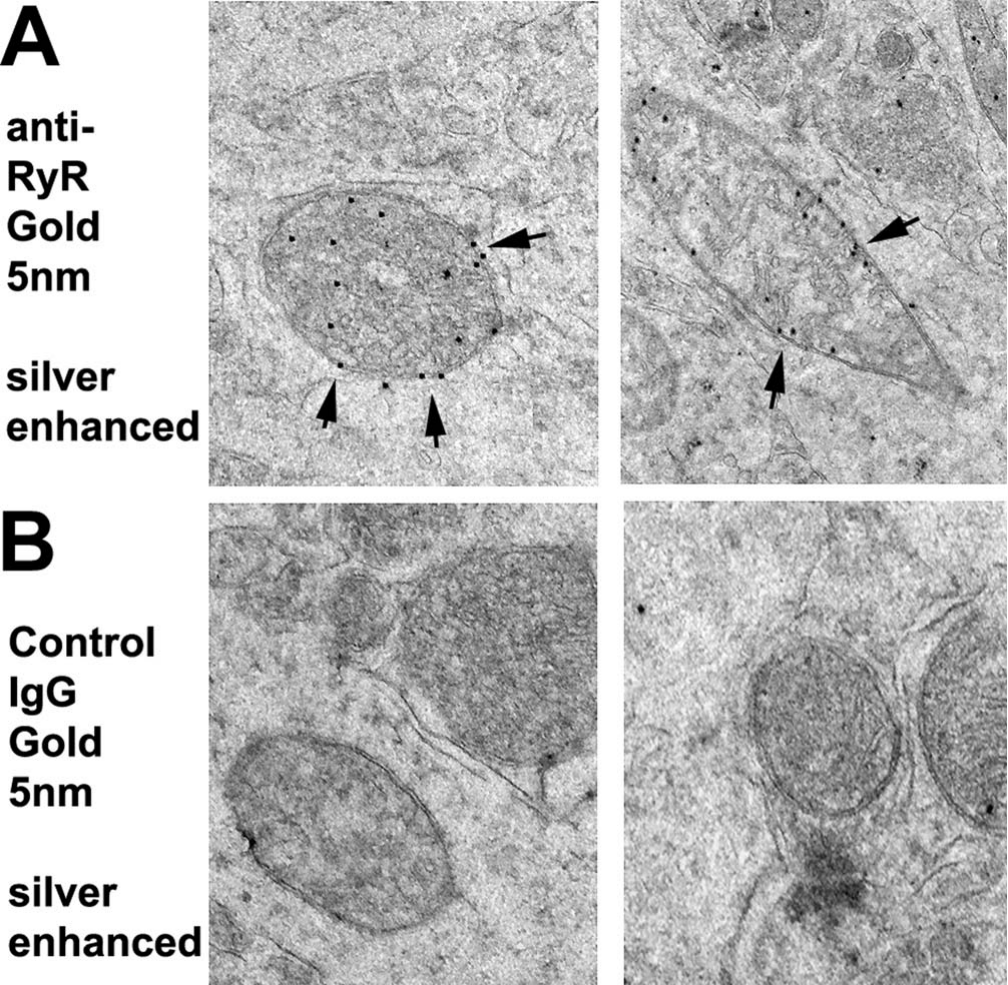
RyR are located in the inner mitochondrial membrane. A, Neuronal RyR expression (short arrows) is contiguous with the inner mitochondrial membranes (IMM) from rodent brain as visualized by silver-enhanced immunogold cytochemistry. B, Control sections were incubated with gold-labeled secondary antibodies in the absence of primary antibody and demonstrate no appreciable, non-specific staining.

The observation that pharmacological antagonism of the RyR in neurons exposed to Tat modulated both mitochondrial membrane potential and Ca^2+^ stores lead us to ask whether we could observe RyR that localize to the mitochondria. To further validate the physiologic significance of this, we used silver-enhanced immunogold immunohistochemistry of thin sections of murine brain tissue to demonstrate that there are RyR present in neuronal mitochondria visualized as distinct punctae that co-localized exclusively with the inner mitochondrial membrane (IMM) (Figure 5A). To rule out non-specific antibody staining, we used a control IgG antibody that demonstrates no significant background staining (Figure 5B).

### Pathologic alterations in ER and mitochondrial morphology in vivo

**Figure 6:**
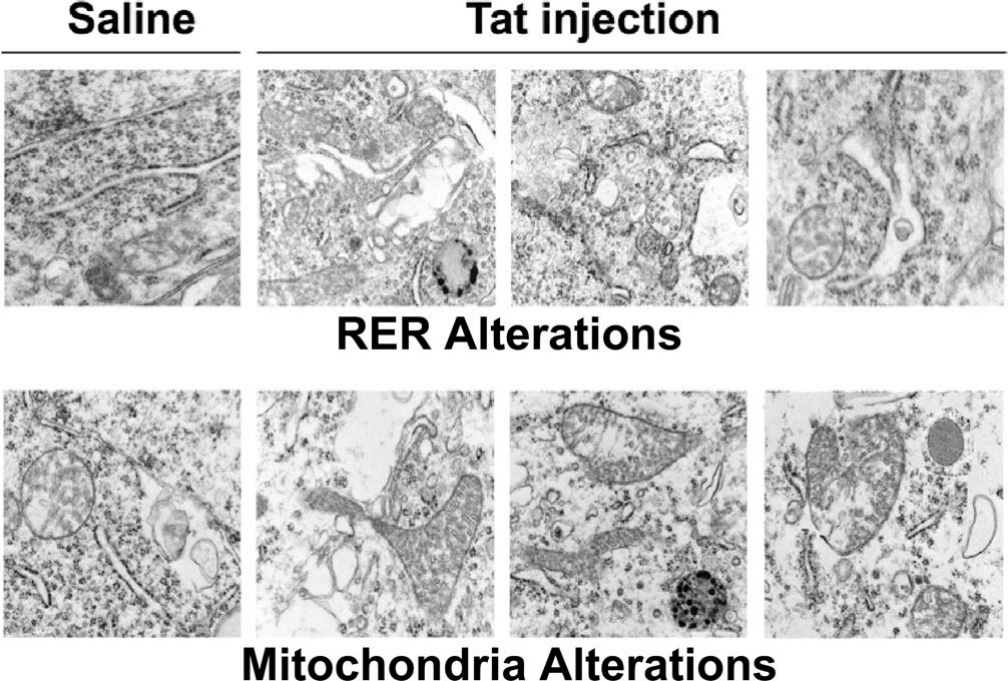
HIV-1 Tat induces endoplasmic reticulum and mitochondrial changes in frontal cortex of mice. Wild type C57Bl/6J received stereotactic injections of vehicle control or HIV-1 Tat (50 µmols) into frontal cortex, followed by sacrifice 4 weeks later. Frontal cortex was processed for electron microscopy. The upper and lower panels on the left depict normal morphology of RER and mitochondria, respectively. The upper panels on the right demonstrate mild ER dilatation with occasional vacuolization. The lower panels on the right demonstrate abnormally enlarged mitochondria with increased cristae.

To determine whether abnormalities in mitochondria and RER morphology also occurred in an *in vivo* model of Tat-induced neurodegeneration, we injected Tat into the frontal cortex of young adult (3 month old) wild type C57Bl/6J mice, and sacrificed them 4 weeks later for ultrastructural analysis. Figure 6 demonstrates that a single injection of Tat had profound, enduring consequences on both RER and mitochondria, in contrast to our in vitro experiments where only RER showed dramatic changes in architecture (Figure 3H). In contrast to mice that received vehicle control injections, Tat-injected mice had dilated ER, with irregularly shaped cisternae and in some cases, vacuolization. Mitochondria were enlarged, irregularly shaped, with abundant cristae.

**Figure 7:**
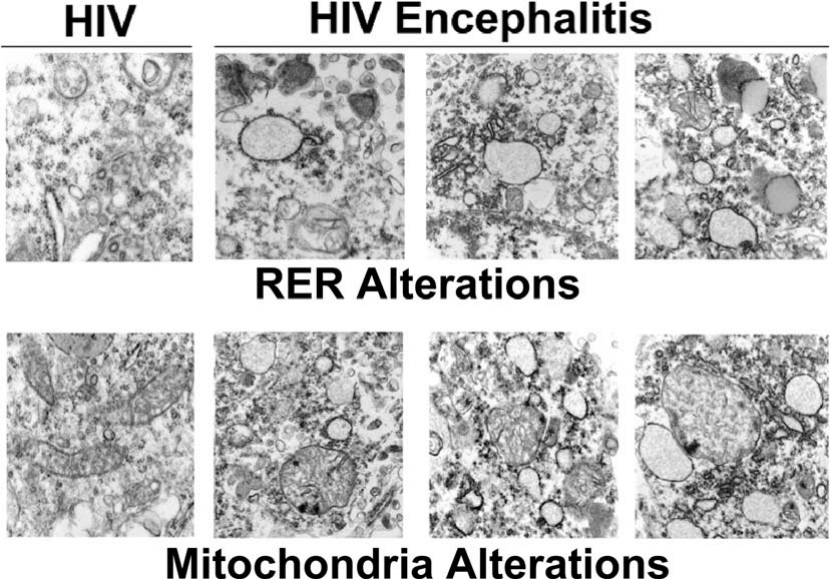
Ultrastructure of frontal cortex from patients with HIVE and dementia. Brain tissue from HIV-1 patients without the neuropathologic hallmarks of HIVE and patients with HIVE and pre-mortem diagnosis of dementia was prepared for electron microscopic analyses as described in Methods. The panels in the montage on the left depict normal neuronal RER and mitochondrial morphology for patients with HIV-1 but no evidence of brain pathology. In contrast, the panels on the upper right depict grossly dilated neuronal RER with scattered deposit of electron dense material and the panels in the lower right depict neuronal mitochondria with abnormal cristae and deposits of electron dense material adjacent to dilated ER cisternae.

Our previous studies [13], [35], [39] have modeled synaptic and mitochondrial pathology that may occur during HIV-1 infection of the CNS. Surprisingly, there has been a paucity of studies investigating ultrastructural changes that occur during HIV-1 induced neurodegeneration, with the exception of a study by Weis et al. [18] that reported vacuolization and thinning of the basal lamina, with an increase in the volume, but not number of cortical vessels in brain tissue from patients with AIDS. To gain a better understanding of how neuronal mitochondria and other subcellular organelles such as ER are affected during HIV-1 neurodegeneration, we performed an ultrastructural survey of frontal cortex from brain tissue of three patients with HIVE and dementia and three patients with HIV-1, but no evidence of brain pathology. Figure 7 demonstrates normal rough ER (RER) cisternae and mitochondria in frontal cortex from an age-matched patient with HIV-1 and no neurologic disease, in contrast to greatly dilated RER (upper panels) with irregularly shaped cisternae and scattered deposits of electron dense material present in frontal cortex of patients with HIVE and dementia. Lower panels depict very abnormal mitochondria with irregular cristae and electron dense material, surrounded by dilated ER cisternae. These findings of pathologic changes in organellar ultrastructure may reflect the chronic effects of HIV-1 infection on normal mitochondrial and RER function in cortical neurons in contrast to our acute *in vitro* model [35], [39]. In our limited survey of three patients with HIVE and three patients with HIV-1 but no discernible brain pathology, we are unable to discern whether HAART is a potential confound because HAART use is present to some degree in both groups.

## Discussion

We have previously shown that HIV-1 Tat has deleterious effects on neuronal calcium homeostasis that initiates a cellular stress response by hyperpolarizing cortical mitochondria [39]. In this work, we provide insights into possible mechanisms for ER and mitochondrial abnormalities observed in the frontal cortices of mice that have received stereotactic injections of Tat and patients with HIVE and dementia. Here we demonstrate that sub-lethal HIV-1 Tat exposure activates the UPR with unique pathologic changes in ER morphology specific to cortical neurons vs. glia (data not shown). In addition to the observed ER effects, our data further suggest that signaling through the RyR plays an integral role in the regulation of mitochondrial homeostasis [39]. The implications of these findings are discussed below.

### The Unfolded Protein Response

The UPR response has been postulated to be a common mechanism for a variety of neurodegenerative disorders due to the observation that unfolded or misfolded protein accumulation may increase during the pathogenesis of these disease states [30]. For example, plaques in Alzheimer's disease (AD) involve the accumulation of β-amyloid and similarly, aggregated α-synuclein is a hallmark of Parkinson's disease (PD), evidence that supports a pivotal role for the UPR in cellular stress [54], [55]. Viral infections have also been implicated in UPR induction, including borna disease virus, flaviviruses (yellow fever, West Nile, etc.) and hepatitis B virus [34], [56], [57]. In this study, we investigated whether Tat could induce the UPR as a key pathogenetic mechanism for neuronal dysfunction that occurs during HIV-1 associated neurologic disease.

The ER is a specialized intracellular organelle whose protein folding capacity is dependent on maintaining a relatively oxidized environment and high calcium concentrations required for chaperone molecules [45]. The ER is responsible for the storage of Ca^2+^ and has the ability to induce rapid efflux of Ca^2+^ in response to a variety of cellular signals, including inositol 1,4,5-triphosphate (IP_3_) receptors and ryanodine receptors (RyR) [40]. HIV-1 Tat is know to modulate intracellular calcium concentrations through several different mechanisms, but these measurements were based on use of calcium sensitive dyes that are not localized to specific intracellular compartments [27]. Using an ER-targeted calmodulin EYFP construct, we were able to ascertain the kinetics of ER calcium modulation by Tat.

We demonstrated that Tat induces a rapid loss of [Ca^2+^] in cortical neuronal ER (Figure 1A), an effect abolished by co-incubation with an antagonist concentration of ryanodine, indicating that there is an interaction of Tat with the RyR but not IP3 receptors (Figure 1B). Tat has several other targets, including ionotropic glutamate receptors [43], [44]. Thus antagonism of either NMDA or non-NMDA receptor subtypes after exposure to a sub-lethal dose of Tat failed to change ER [Ca^2+^] (Figure 1C). The data suggests one of two possible mechanisms: either Tat is activating the RyR through a direct interaction, or it is sensitizing the receptor to cytosolic calcium; a similar effect can be observed with caffeine application [40].

We next investigated what sort of response associated with the UPR may occur in our experimental paradigm. As unprocessed proteins accumulate in the ER a repertoire of cellular defense pathways are activated to restore proper function. An early event in UPR induction is the phosphorylation of one or more, transmembrane proteins that relay cytosolic information to the ER [30]. Activation of the transmembrane kinase IRE1 pathway occurs after it dimerizes and autophosphorylates, activating an RNase domain [46], [47]. Another parallel pathway that is metabolically active during the UPR involves PERK phosphorylation (p-PERK). p-PERK perpetuates the UPR by phosphorylating the eukaryotic initiation factor eIF2α (p-eIF2α). Because the immediate consequence of increased expression of p-eIF2α is a whole scale shutdown of protein translation in the ER, we investigated the phosphorylation of these protein species.

We demonstrate that IREα and PERK phosphorylation occurred in our paradigm because of the increase in abundance for p-PERK and phosphorylated eIF2α (Figure 2A, B) while simultaneously, p-IREα up-regulated the gene XBP1 that splices a 26nt [32] from the mRNA to produce the transcription factor product, XBPs (Figure 2A, B). Interestingly, the Tat induced upregulation of p-PERK, XBP_u_, and XBP1_s_ was significantly attenuated (to 90% of Tat expression) by the pretreatment of ryanodine [20 µM] for 30 min before treatment at the 1-hour time point (Supplemental Figure S1). Similarly, the increase in p- eIF2α expression was attenuated at the 6-hour time point (Supplemental Figure S1). Up-regulation of XBPs, a crucial transcription factor, can increase the capacity of the ER to fold proteins during the UPR that could be attributed to the activation of this portion of UPR pathways [29], [46]. The discrepancy between the small magnitude of the protein changes relative to the striking ultrastructural changes observed after 8 nM Tat treatment in cortical neurons (Figure 3E–H) suggests that there may be some type of amplification process via post-translational modification of cytoskeletal proteins that control shape of ER cisternae. Additionally, the lack of changes in mitochondrial morphology (i.e. cristae) during acute exposure to Tat in our in vitro model, suggests that this phenomenon is dependent on pathways that subserve an inflammatory response only present in an *in vivo* milieu. While the ability of antagonist doses of ryanodine to inhibit Tat-induced phosphorylation of UPR gene products confirms that the rapid decrease in ER [Ca^2+^] is the initiating step for ER dysfunction, further studies beyond the scope of this report are required to delineate the mechanism(s) responsible for these morphologic changes and resolve the kinetic differences between our *in vitro* and *in vivo* models.

Regardless, the most striking feature of the Tat-induced UPR is the morphological changes that are apparent in the EM photomicrographs (Figure 3). In untreated neurons, the ER is barely visible, as rows of ribosomes that line the ER membrane (Figure 4A, C, D). This normal ER morphology is disrupted when Tat induces the UPR and as a result, the ER becomes dilated, with detached ribosomes no longer in apposition to the ER membrane (Figure 3B, G, H). There is also a structural abnormality that we were unable to identify and appears to be a filament running “through” the ER (Figure 3B).

### Downstream Consequences of UPR Induction

The observed ER dysfunction and induction of the UPR pathway in cortical neurons may have additional ramifications. HIV infected macrophages or restrictively infected astrocytes release TNFα into the extracellular milieu and is a key inflammatory mediator in models for the observed chronic inflammation in HIV Associated Neurologic Disease (HAND) [2], [58]–[60]. Interestingly, induction of the UPR intersects with the TNFα signaling pathway because the phosphorylated species of the transmembrane protein IREα can interact directly with the tumor necrosis factor receptor-associated factor 2 (TRAF2), which is also an initiation step for induction of the UPR [61], [62].

### Mitochondrial hyperpolarization and the RyR

Perhaps our most intriguing data demonstrates the presence of a functionally active RyR proximate to or physically associated with mitochondria. We previously examined whether loss of mitochondrial calcium was responsible for mitochondrial hyperpolarization [39], and demonstrated there is a coordinated loss of mitochondrial calcium with membrane hyperpolarization (Figure 5 in Ref. 39). Based on this, we pre-treated cortical neurons with an antagonist concentration of ryanodine and then measured [Ca^2+^]_mito_ and ΔΨ_m_ in response to Tat (Figure 4). Ryanodine attenuated Tat's effects on both [Ca^2+^]_mito_ and ΔΨ_m_, suggesting that there is a functional RyR on mitochondria that is responsible for Tat's ability to reduce [Ca^2+^]_mito_ and hyperpolarize the mitochondrial membrane, a novel finding that has not been previously demonstrated in other studies investigating expression of RyR in mitochondrial membranes and its implications in neurodegenerative diseases [51], [52]. In addition to the pharmacological evidence for a functional RyR on mitochondria, we demonstrate the physical presence of mitochondrial RyR localized to the IMM, using silver-enhanced immunogold electron microscopy (Figure 5). Unlike previous studies, we performed these EM studies on intact neurons in murine brain rather than on isolated mitochondria, which eliminates the confounding factor of ER contamination after subcellular fractionation [51], [52]. Because the outer mitochondrial membrane is very porous in contrast to the IMM, it is highly likely that this population of RyR represents a new therapeutic target for processes that alter mitochondrial calcium homeostasis.

In summary, our data implicates multiple roles for a sub-lethal dose of HIV-1 Tat in eliciting a general stress response in cortical neurons that involves activation of the RyR with reversible ER dysfunction, the UPR and mitochondrial hyperpolarization. These results raise the interesting and novel possibility that the RyR may be a crucial target for neuroprotection in HIV-associated neurologic disease.

## Materials and Methods

### Reagents

The recombinant HIV-1 Tat_1-72_ was generously provided by the laboratories of Dr. Avindra Nath and Phil Ray [43]. The D1ER construct was a generous gift from Dr. Roger Y. Tsien (Dept. of Pharmacology, UCSD, San Diego, CA) and the YC3.1 construct [53], [63] was a generous gift from laboratory of Dr. Nicolas Demaurex (Dept. of Physiology, University of Geneva, Switzerland) and was originally developed by Dr. Roger Y. Tsien (Dept. of Pharmacology, UCSD, San Diego, CA). The dyes rhodamine 123, and ER-Tracker Red were purchased from Molecular Probes (Invitrogen, Carlsbad, CA). B27 supplement (with and without anti-oxidants), Neurobasal media and PCR primers were purchased from Invitrogen. Ryanodine was purchased from Calbiochem (EMD Biosciences, La Jolla, CA) and dantrolene was purchased from Tocris (Ellisville, MO). Antibodies for western blots were purchased from Santa Cruz Biotechnology (Santa Cruz, CA). All other chemicals and reagents were purchased from Sigma (St. Louis, MO).

### Primary Neuronal Cell Culture

Primary neuronal cortical cultures were harvested and prepared from embryonic day 18 Sprague-Dawley rat pups as previously described by Brewer [64] and modified in Norman et al. [39]. In brief, the cortices were isolated from a litter of E18 rats and the meninges and extraneous tissue removed. The cortices were incubated in 2mL of Ca^2+^/Mg^2+^ free Hank's balanced salt solution (HBSS with 10 mM HEPES, pH 7.3) with gentamicin (50 µg/mL) and 0.25% trypsin for 15 min at 37°C. The cells were centrifuged at 1000 rpm for 5 min, washed twice with HBSS (with Ca^2+^/Mg^2+^), then dissociated in Neurobasal media supplemented with glutamate, gentamicin and B27 supplement (Life Technologies, Gaitherburg, MD) by 10 passages through a 0.9 mm bore pipette tip. Dissociated cells were counted using the trypan blue viability assay and were plated on poly-D-lysine coated cell culture plastic and incubated in a humidified atmosphere of 5% CO_2_/95% air at 37°C. The supplemented Neurobasal media is modified for an anti-oxidant free culture as described by Perry et al. [35], and inhibits the growth of glial cell populations. The resulting cultures are 98% pure neuronal cultures [64]. Cultures were used for experiments at days *in vitro* (DIV) 11-14 unless otherwise noted.

### Animal studies

Male wild type C57Bl/6J mice, three months old, received stereotactic injections (AP −1.5 mm, lateral 1 mm, depth 2 mm) of 50 µmols of Tat_1-72_ or control vehicle in 2 µl, followed by sacrifice and intracardiac perfusion with paraformaldehyde 4 weeks later. All animal studies were conducted under the NIH guidelines of the committee for animal resources of UCSD.

### Electron Microscopy

#### In vitro studies

Electron microscopy was performed using the cultured cell pop-off method described in de Mesy Jensen, et al. [65]. Briefly, cells were fixed onto chamber slides using 0.1 M phosphate buffered 2.5% glutaraldehyde, pH 7.4 overnight. The slides were rinsed in two changes of phosphate buffer and post-fix using phosphate buffered 1.0% osmium tetroxide for 30 minutes. Cells were dehydrated by passing slides through a graded series of ethanol to 100% (×3), transitioning into Spurr epoxy resin, then 100% resin overnight. Embedment of cells was accomplished after filling size 3 Beam capsules with fresh resin and inverting for placement upon glass slide surface. The slides/capsules were polymerized at 70°C overnight. The next day, the surface tension between capsules and slide surfaces was broken by dipping slides into liquid nitrogen several times until capsules disengaged. Inverted capsules were examined under the light microscope and an area to be thin-sectioned was chosen. The capsules were trimmed of excess epoxy and thin sectioned onto copper grids. The grids were stained with aqueous uranyl acetate and lead citrate. The cells were examined with a Hitachi 7100 transmission electron microscope with digital image capture using a MegaView III camera (Soft-Imaging System, Lakewood, Colorado).

#### Ex vivo studies

Frontal cortices from three separate cases with the neuropathologic hallmarks of HIV encephalitis (HIVE) and a pre-mortem history of HIV-1 associated dementia (HAD) or age-matched HIV-1 patients without discernible evidence of HIVE or HAD were used in these studies. The pre-mortem history for these patients revealed that 2/3 cases with HIVE had not received HAART for >5 months prior to death, while the other case had HAART 20 days prior to death. 2/3 of the cases without evidence of HIVE were on HAART until 1 week prior to death and the other case discontinued HAART 2.5 months prior to demise. Brain tissue regions from these patients were prepared for electron microscopic studies by vibratoming 40 µM sections from paraformaldehyde-fixed tissue blocks, followed by embedment in Epon-Araldite and sectioning with the Reichert Ultracut-E ultramicrotome (Leica, Vienna, Austria), placement on 200-mesh copper grids, and staining in saturated ethanol/uranyl acetate and bismuth nitrate, as previously described [66]. From each case, approximately 10 sections (90-nm-thick) were analyzed with the Zeiss EM10 Electron Microscope at 5, 10, 20, and 50 K magnifications in order to evaluate the ultrastructural characteristics of subcellular organelles in neurons. For animal studies, mice were sacrificed and paraformaldehyde-fixed tissue blocks were cut by vibratome at 40 µM thickness, followed by post-fixation processing and analysis as described above.

### Immunogold Cytochemistry

Briefly, Vibratome sections were fixed in 0.25% glutaraldehyde and 3% paraformaldehyde in 0.1 M cacodylate buffer (pH 7.4), and then pre-embedded with 50%Durcupan epoxy resin, and 50% ethanol (anhydrous) for 30 min, and then embedded in Durcupan mix epoxy resin with polymerization under vacuum at 60 C for 48 hrs. After the resin was polymerized, tissues were mounted into plastic cylinders, sectioned with an ultra microtome (Reichert Ultracut E) at 60 nm thickness and collected on nickel grids for immunogold labeling. The grids were treated for antigen retrieval (sodium periodate saturated in water) for 1 min, washed in water, blocked with 3% BSA in TBS for 30 min, and then incubated with the anti-RyR (Sigma, Clone 34C; 1∶100) overnight. The following day, the grids were washed in TBS, blocked with 3% BSA, and incubated with the secondary antibody, IgG-antimouse/10 nm gold particles (AURION Immunogold reagents) for 2 hrs. at room temp. The grids were washed in TBS and distilled water. To enhance RyR labeling, we used a silver mixture (AURION R-gent SE-EM) for 25 min, then washed extensively with distilled water, and contrasted as follows: The immunostained grids were post-stained using saturated Uranyl Acetate solution in 50% Ethanol, for 20 min. at room temperature, then washed in distilled water, and placed in bismuth nitrate solution for 10 min followed by a final wash in distilled water. In control experiments, grids were incubated with gold-labeled secondary antibodies in the absence of primary antibody. The immunolabeled grids were analyzed with a Zeiss EM10 electron microscope; neurons were identified morphologically and the electron micrographs were obtained at a magnification of 35,000.

### Image Capture and Image Analysis

In order to image and measure physiological changes in real time, we employed fluorescent dyes. The protocol for fluorophore was utilization was previously described (MY). Briefly, samples were placed on a DC60 warming stage (Linkam Sci. Instruments, Surrey, UK) and maintained at 37°C for the duration of the experiment. A single field was monitored during the course of the experiment and images were taken using an Olympus IX-70 microscope with 40× objective and an Apogee KX32ME CCD camera. The resulting images were analyzed using Scanalytics IPLab software. Quantification of the neuronal fluorescent intensity was determined by the total sum of the recorded pixel values within the specified region of interest (ROI), for each image series captured.

### Internal Endoplasmic Reticulum and Mitochondria Ca^2+^ Measurements

Endoplasmic reticulum and mitochondrial Ca^2+^ measurements were accomplished through the use of either an ER targeted or a mitochondrial targeted YFP-calmodulin construct. Briefly, cortical neurons were transfected using Lipofectamine 2000 (Invitrogen, Carlsbad, CA) using 2 µL Lipofectamine to 1 µg DNA for each culture plate. Cortical neurons were incubated for 24 hours at 5% CO2/95% air at 37°C for before the Lipofectamine was removed and returned to the incubator for 4 days. Transfected cultures were placed in Leibovitz's L-15 medium (Gibco) and incubated at 37°C in room air for 25 minutes before imaging. The appropriate experimental treatment was added directly to the media and the fluorescence resonance energy transfer (FRET, i.e. whereby the donor fluorophore CFP excites the acceptor fluorophore EYFP via nonradiative dipole-dipole interaction) measured from cyan fluorescent protein (CFP) at 425/480 (ex/em) (custom filter; Chroma Technology) to EYFP fluorescence at 425/540 (ex/em) (Lucifer Yellow filter; Chroma Technology) [34], [41]. Data are expressed as the ratio between the emissions of CFP to EYFP at 480/540 nm.

### Assessing mitochondrial ΔΨ_m_

Mitochondrial ΔΨ_m_ was determined, as described previously [24], [39]. Briefly, 10 µM rhod123 was incubated with cortical neuronal cultures under normal incubation conditions for 30 min. The rhod123-containing medium was removed, and the cultures were placed in CO2-insensitive, pH-stable Leibovitz's L-15 medium (Invitrogen Life Technologies) containing 10 µM rhod123 and incubated at 37°C in room air for 15 min to allow for the rhod123 fluorescent signal to reach a steady state. Culture treatment was applied directly to the bath medium. Samples were imaged using a Texas Red filter (Chroma Technology) at 560/645 (ex/em). The high concentration of rhod123 causes the fluorophore to quench, which inversely correlates with the ΔΨm.

### Immunoblot analysis

Primary cortical neurons (1×10^6^) were rinsed three times with ice-cold phosphate buffered saline (PBS) and lysed in lysis buffer (50 mM Tris-HCl, pH 7.4, 100 mM NaCl, 5 mM EDTA, 1 mM dithiothreitol, 1% Nonidet P-40, 0.1 mM sodium vanadate, 20 mM b-glycerophosphate, and 20 mM p-nitrophenylphosphate) in the presence of 1 mM phenylmethylsulphonylfluoride (PMSF) and a protease inhibitor cocktail (Sigma, St. Louis, MO). Samples were incubated on ice for 30 min, homogenized, and centrifuged for 5 min at 13,000g at 4°C. The pellet was discarded and the supernatant total protein was quantified using the Lowry protocol (Dc Protein Assay, Bio Rad, Hercules, CA). An equal amount of protein (25 µg) was used for each sample and they were separated by 12% SDS-PAGE. The protein was transferred onto a polyvinylidene difluoride (PVDF) membrane. The resulting membrane was blocked with a 5% milk solution (0.3% Tween-PBS) for one hour at room temperature. The following antibodies were used to probe the membrane: PERK (1∶500, anti-rabbit), p-PERK (1∶1000, anti-rabbit), eIF-2α (1∶1000, anti-goat), p-eIF-2α (1∶500, anti-goat), and XBP1 (1∶500, anti-rabbit). In addition, each blot was probed with α-tubulin (1∶2000, anti-mouse) as a loading control, and densitometric data were normalized to α-tubulin. The resulting bands were detected using chemiluminescence (ECL Western Blotting Detection, Amersham, Buckinghamshire, UK). Western blots were digitally photographed using an illuminator with a digital camera (Northern Light, Canada) and densitometry was performed using Scion Image.

### Data and statistical analysis

The raw data were analyzed from 3–5 independent experiments and expressed as the mean±SEM. Where applicable, the percentage of control and percentage of control SEM for each treatment condition were calculated by dividing the raw means and raw SEM's by the control condition raw mean. A Student's t test using a two-tailed distribution and unequal variance was used to compare data. A probability of p<0.05 was considered statistically significant.

## Supporting Information

Figure S1 Treatment with Ryanodine decreases acute UPR pathway induction. A, Cortical neurons were either pretreated with an antagonist concentration of ryanodine [20 µM] for 30 min before Tat [8 nM] exposure or were exposed to Tat alone and UPR protein levels were measured via western blotting. The bands shown are representative of all western blots quantified. B, Densitometry was performed as described in the Methods section, but bands were normalized to expression of beta tubulin (BT), which was invariant throughout the time course of the experiment. Ryanodine bands were expressed as the percent optical density (O.D.) of the Tat only-treated bands. (n = 3, * = p<0.05 for XBPu, XBPs and p-PERK @1 hr and for XBPu, XBPs and p-eIF2α @6 hr).
(0.94 MB DOC)
