# Supplementary figures and images for: HIV-1 Tat Activates Neuronal Ryanodine Receptors with Rapid Induction of the Unfolded Protein Response and Mitochondrial Hyperpolarization
Source: PLoS One. 2008 Nov 14;3(11):e3731. doi: 10.1371/journal.pone.0003731 (PMC2579580; doi:10.1371/journal.pone.0003731)

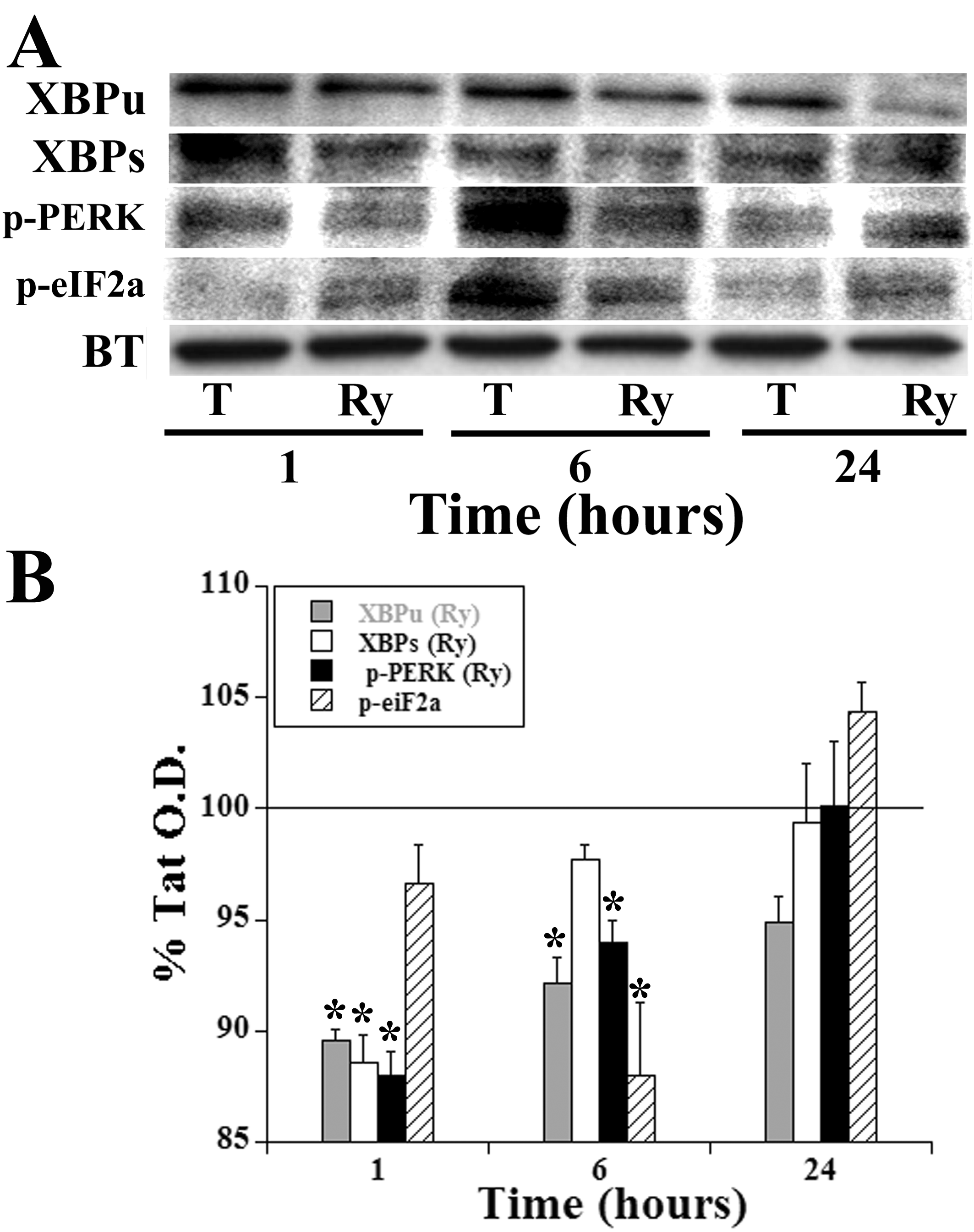

Supplement: Figure S1 — Treatment with Ryanodine decreases acute UPR pathway induction. A, Cortical neurons were either pretreated with an antagonist concentration of ryanodine [20 µM] for 30 min before Tat [8 nM] exposure or were exposed to Tat alone and UPR protein levels were measured via western blotting. The bands shown are representative of all western blots quantified. B, Densitometry was performed as described in the Methods section, but bands were normalized to expression of beta tubulin (BT), which was invariant throughout the time course of the experiment. Ryanodine bands were expressed as the percent optical density (O.D.) of the Tat only-treated bands. (n = 3, * = p<0.05 for XBPu, XBPs and p-PERK @1 hr and for XBPu, XBPs and p-eIF2α @6 hr). (0.94 MB DOC) [file pone.0003731.s001.doc]
